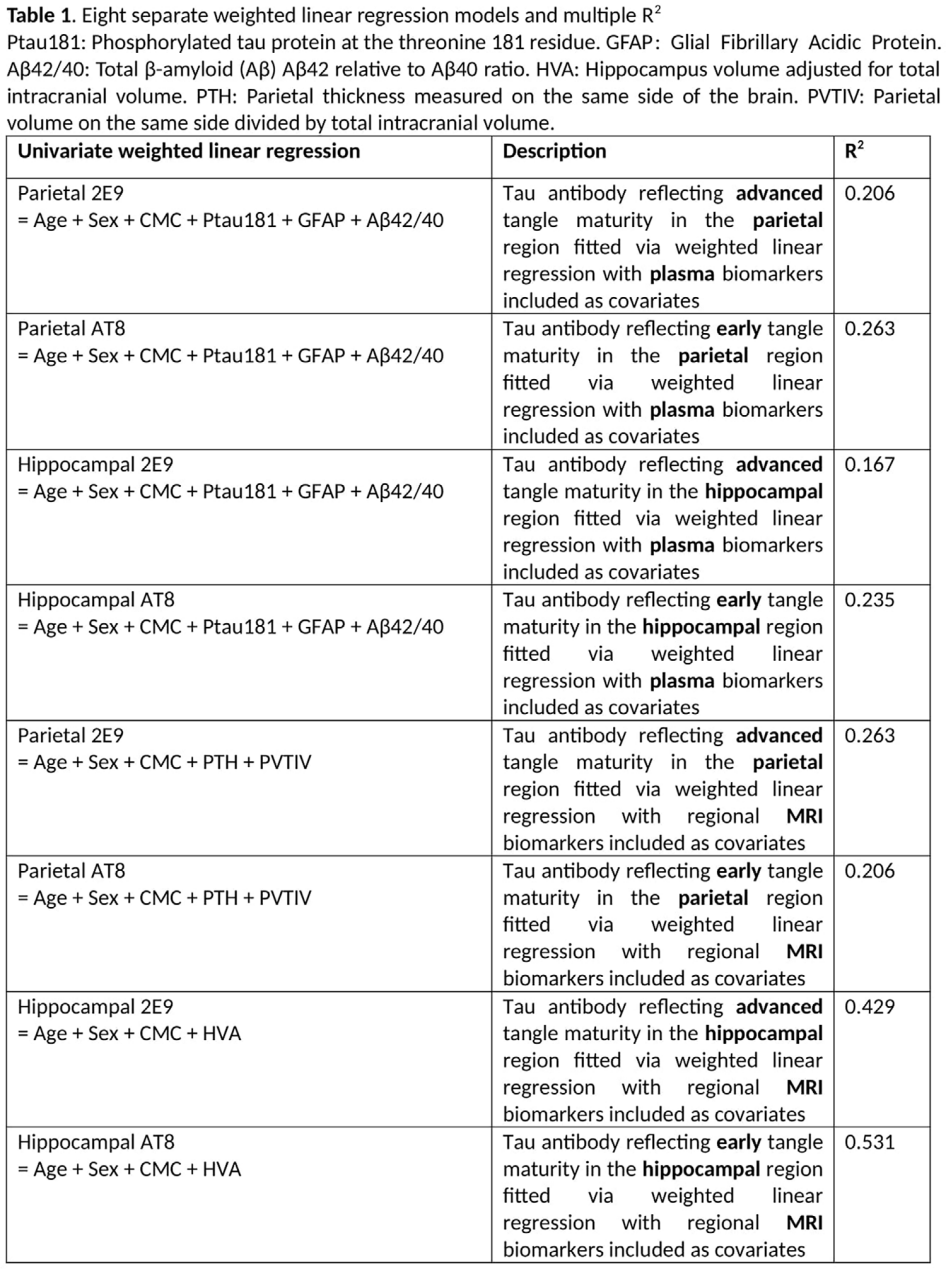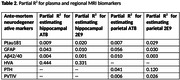# Plasma and MRI biomarkers predict post‐mortem tau pathology

**DOI:** 10.1002/alz.093893

**Published:** 2025-01-09

**Authors:** Mingzhao Hu, Christina M. Moloney, Scott A. Przybelski, Alicia Algeciras‐Schimnich, Angela J. Fought, Darren M. Rothberg, Aivi T. Nguyen, Ross R. Reichard, Dennis W. Dickson, David S. Knopman, Clifford R. Jack, Ronald C. Petersen, Michelle M. Mielke, Jonathan Graff‐Radford, Melissa E. Murray, Prashanthi Vemuri

**Affiliations:** ^1^ Department of Quantitative Health Sciences, Mayo Clinic, Rochester, MN USA; ^2^ Department of Neuroscience, Mayo Clinic, Jacksonville, FL USA; ^3^ Mayo Clinic, Rochester, MN USA; ^4^ Department of Laboratory Medicine and Pathology, Mayo Clinic, Rochester, MN USA; ^5^ Department of Neurology, Mayo Clinic, Rochester, MN USA; ^6^ Wake Forest University School of Medicine, Winston‐Salem, NC USA; ^7^ Mayo Clinic, Jacksonville, FL USA

## Abstract

**Background:**

Imaging and plasma markers are used as key indicators of disease for Alzheimer’s disease (AD) but their usefulness in predicting regional tau pathology is relatively understudied. Our objective was to construct predictive models for regional tau pathology measured on postmortem brain tissue using multiple ante‐mortem AD biomarkers. We focused on hippocampal and parietal regions that were immunostained with AT8 and 2E9 that reflect early and advanced aspects of tangle maturity, respectively.

**Methods:**

Using autopsy data from 63 participants enrolled in the Mayo Clinic Study of Aging (MCSA) where the last clinical visit is within three years of death, we analyzed three ante‐mortem plasma markers ‐ phosphorylated tau protein at the threonine 181 residue, glial fibrillary acidic protein, total ß‐amyloid Aß42 relative to Aß40 ratio (Quanterix Simoa HD‐X Analyzer), and three regional MRI markers computed using SPM12 ‐ parietal thickness, parietal volume relative to total intracranial volume ratio, and hippocampal volume adjusted for total intracranial volume. Based on eight separate cross‐sectional linear regression models weighted by the inverse of time from last visit to death, we estimated the multiple and partial R2 predicting post‐mortem pathology measures, including AT8 and 2E9 in both the hippocampal and parietal regions. All models were adjusted for age, sex, and cardiovascular and metabolic conditions score.

**Results:**

The MRI models (R2 of 0.26‐0.53) had greater predictive power compared to the plasma models (R2 of 0.17‐0.26). The best multiple R2 was 0.53 for prediction of hippocampal AT8 using antemortem hippocampal volume adjusted for total intracranial volume followed by R2 of 0.43 for prediction of hippocampal 2E9 using hippocampal volume adjusted for total intracranial volume. Though the model prediction of parietal AT8 and 2E9 measurements was low (R2 of 0.21‐0.26), parietal thickness exhibited better predictive performance than parietal volume relative to total intracranial volume ratio.

**Conclusions:**

The data we presented here may be utilized to validate biomarkers using regional pathology. Our results support that regional MRI is sensitive to tau pathology. Newer MRI methods should be tested to examine the specificity of newer quantitative measurements to tau pathology.